# Neurological damages in COVID‐19 patients: Mechanisms and preventive interventions

**DOI:** 10.1002/mco2.247

**Published:** 2023-04-06

**Authors:** Sibani Sarkar, Subhajit Karmakar, Malini Basu, Pratyasha Ghosh, Mrinal K Ghosh

**Affiliations:** ^1^ Division of Cancer Biology and Inflammatory Disorder Signal Transduction in Cancer and Stem Cells Laboratory Council of Scientific and Industrial Research‐Indian Institute of Chemical Biology (CSIR‐IICB) Kolkata India; ^2^ Department of Microbiology Dhruba Chand Halder College, University of Calcutta Dakshin Barasat WB India; ^3^ Department of Economics Bethune College University of Calcutta Kolkata India

**Keywords:** antioxidant, COVID‐19, ischemia, neurological damage, prevention therapy, ROS, SARS‐CoV

## Abstract

Severe acute respiratory syndrome coronavirus 2 (SARS‐CoV‐2), a novel coronavirus, causes coronavirus disease 2019 (COVID‐19) which led to neurological damage and increased mortality worldwide in its second and third waves. It is associated with systemic inflammation, myocardial infarction, neurological illness including ischemic strokes (e.g., cardiac and cerebral ischemia), and even death through multi‐organ failure. At the early stage, the virus infects the lung epithelial cells and is slowly transmitted to the other organs including the gastrointestinal tract, blood vessels, kidneys, heart, and brain. The neurological effect of the virus is mainly due to hypoxia‐driven reactive oxygen species (ROS) and generated cytokine storm. Internalization of SARS‐CoV‐2 triggers ROS production and modulation of the immunological cascade which ultimately initiates the hypercoagulable state and vascular thrombosis. Suppression of immunological machinery and inhibition of ROS play an important role in neurological disturbances. So, COVID‐19 associated damage to the central nervous system, patients need special care to prevent multi‐organ failure at later stages of disease progression. Here in this review, we are selectively discussing these issues and possible antioxidant‐based prevention therapies for COVID‐19‐associated neurological damage that leads to multi‐organ failure.

## Graphical abstract

A schematic overview of severe acute respiratory syndrome coronavirus 2 mediated neurological damages in coronavirus disease 2019 causing cerebral ischemia.

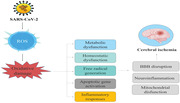

## 1 INTRODUCTION

Severe acute respiratory syndrome coronavirus 2 (SARS‐CoV‐2) in coronavirus disease 2019 (COVID‐19) has been described as a pandemic by World Health Organization (WHO) in March 2020 due to its quick spread to many countries.^1^ The notable symptoms of COVID‐19 are fever, cough, and fatigue as well as hemoptysis and dyspnea. It is an enveloped RNA virus that belongs to the family of Coronaviridae.^2^ It was reported that severe pneumonia occurred during previous SARS‐CoV disease in 2003 and pulmonary alveolar collapse happened within a few hours of infection leading to impaired oxygen exchange. Common clinical symptoms of COVID‐19 include high respiratory frequency, dyspnea, abnormal partial pressure of arterial oxygen to fraction of impaired oxygen ratio (<300), low blood oxygen saturation (<93%), high lymphopenia, high lactate dehydrogenase, creatine kinase, production of cytokines, secretion of chemokines, and quickly generate a cytokine storm of both pro‐inflammatory and anti‐inflammatory types.^3^, ^4^ In COVID‐19, SARS‐CoV‐2 can easily spread to multiple tissues^5^ and organs in the human body and WHO guidelines assess the severity of the pandemic by the impact of the disease.^6^, ^7^ The human‐to‐human spreading of the virus reflects rapid communicability and spread of the disease.^8^ The most preferred targets in the human body are the upper respiratory tract and lungs.^9^

Oxidative stress and the immune system play important roles in disease severity including neuronal cell damage. Cyclic changes in oxygen levels majorly produce reactive oxygen species (ROS), a major factor for causing metabolic and physiologic alterations in COVID‐19 patients.^10^, ^11^ Respiratory system in COVID‐19 patients also initiates hypoxia in the brain that triggers ROS production and contributes to tissue damage. Moreover, immunity and inflammation are the key elements of disease biology which are occasionally linked with neurological disorders and ischemia‐related deaths. Pro‐inflammatory molecules interleukin (IL)‐1 and IL‐6 are also increased in COVID‐19 resulting in the rupture of atherosclerotic plaques which leads to acute lung damage.^12^ Nevertheless, immunomodulation is not fully characterized yet as a deleterious effect, but proper guidance and participation of T helper and T effector cells are necessary to harness the therapy against COVID‐19. In recent times, several documents suggest that both CD4^+^ and CD8^+^ T cells are involved in COVID‐19‐mediated ischemic brain injury where self‐reactive T cells can attack the brain tissues due to cytokine response resulting in a reduced number of monocytes, upregulation of the components of anti‐inflammatory circuits, impairment of some effector proteins leading to long term damage of the brain and ultimately cause the death of the patients.^13^, ^14^

The brain is protected from pathogens due to the presence of the Blood Brain Barrier (BBB) and cerebrospinal fluid (CSF) barrier. Moreover, immune receptors in the brain cells can detect pathogens to recruit leukocytes in the central nervous system (CNS).^15^, ^16^, ^17^ However, inflammatory immune responses increase the BBB permeability which facilitates the entry of SARS‐CoV2 leading to life‐threatening neurological damage. After transmission of SARS‐CoV‐2 to the brain, it causes the development and prognosis of neurological disorders and cerebral diseases. In severe cases, patients develop acute respiratory distress syndrome and cerebral problems with slow and sustained failure of one or more organs.^18^ Different pathophysiological processes like cytokine production, inflammation, and cell death occur in COVID‐19 patients. In the ischemic signaling cascade, various molecules are taken to control the inflammatory signaling and make an impact on different phases of the ischemic damage through innate and adaptive immune responses.^19^

When transmission of SARS‐CoV‐2 occurs from the respiratory system to the CNS, it causes symptoms like headache, anosmia, dyspepsia, dizziness, and impaired consciousness.^20^, ^21^ Occasionally the severely affected patients also suffer from cerebrovascular diseases due to neurological damages especially ischemic stroke caused by uncontrolled inflammation, immobilization, hypoxia, and diffuse intravascular coagulation.^22^ So, coagulopathy and vascular endothelial dysfunctions are the major complications of COVID‐19.^23^ Infection in the brain cannot be properly treated because of the presence of BBB through which large drug molecules cannot easily pass. Here in this report, authors critically address the biochemical changes due to oxidative stress, inflammatory immune response, and possible therapeutic strategies to prevent neurological problems in COVID‐19. As proper drugs are not available in the market for COVID‐19‐specific treatment, only possible supportive and preventive therapies are prescribed by the doctors along with the repurposing of anti‐viral and anti‐inflammatory drugs. Thus, in the present study, the use of antioxidants as adjuvants for neurological manifestations of COVID‐19 patients is also highlighted at the end.

## 2 TRANSMISSIONS OF THE SARS‐COV‐2 VIRUS TO THE BRAIN

**FIGURE 1:**
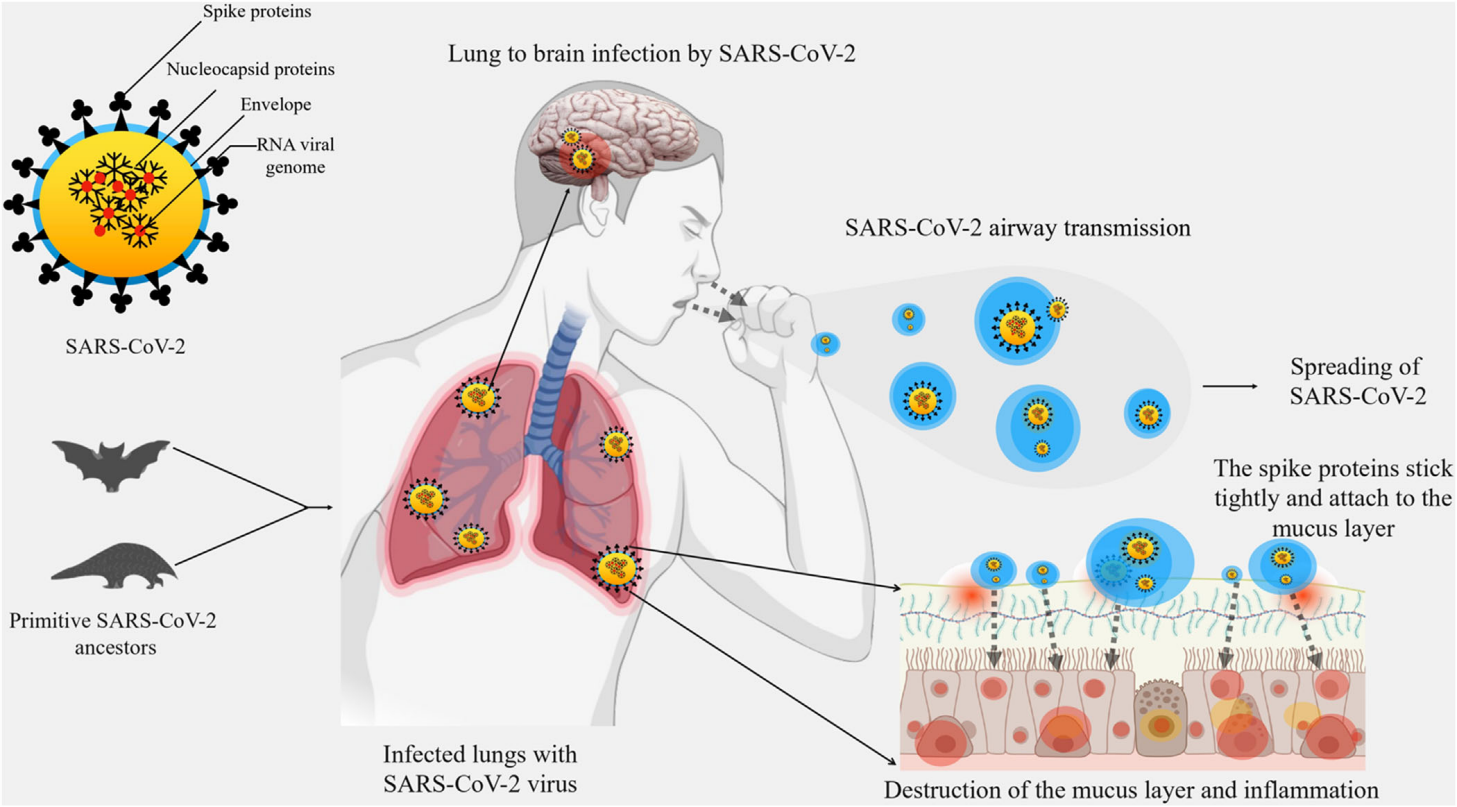
Severe acute respiratory syndrome coronavirus 2 (SARS‐CoV‐2): Mode of transmission. Figure depicts transmission from ancestral source, airway transmission, spreading, and lung to brain transmission (*use of Biorender as tools to generate the figure*).

The major route of transmission of SARS‐CoV‐2 is through air droplets and the person's direct contact.^24^, ^25^ SARS‐CoV‐2 can enter susceptible epithelial cells in an independent endosomal pathway. Additionally, the virus is attached to the host cells' receptors followed by recognition, cleavage by proteases, and fusion with proteins. Finally, it enters the host cells by binding its transmembrane spike (S) glycoproteins to the cell‐specific receptors, for example, angiotensin‐converting enzyme‐2 (ACE2).^26^ The interaction cascade plays a prominent role primarily in transmission and pathogenicity (Figure 1). Next, the Zn‐metallopeptidases convert Angiotensin II to Angiotensin I to VII.^27^ Interaction of ACE2 with Chromatin licensing and DNA replication factor 1 (CTD1) (the receptor binding domain [RBD]) increases its efficiency for spreading the infection and transmission.^28^, ^29^ Binding of ACE2 to different configurations of CTD1 controls the interaction between CTD1, CTD2, S1 subunit of ACE2 complex and its S2 subunit. The surface exposed glycoprotein S comprised of two main functional subunits which are the major components for the binding, fusion of the virus, and entry into the target cells.^30^ The RBD of the S1 subunit has an affinity for binding to the host receptor which differs in different COVs and decides the infection and disease severity.^31^ The S2 subunit is responsible to fuse the virus with the host cell membrane.^32^, ^33^ The crucial steps are proteolytic priming of the S protein to fuse the virus with the host cell membrane followed by delivering the fusion peptide by host proteases and finally the transfer of SARS‐CoV‐2 RNA into the host cell's cytoplasm.^34^, ^35^ The infectivity of SARS‐CoV‐2 is increased by ∼30 folds and possesses a higher furin score than the previous SARS‐CoV.^36^ SARS‐CoV‐2 infection takes place through the involvement of ACE2 which is predominantly overexpressed in the epithelial layer of the lungs.^37^, ^38^ Infection occurs first in the lungs and then transmits to the heart, brain, kidney, intestine, and so forth. Acute pulmonary embolism, deep vein thrombosis, ischemic stroke, and myocardial infarction have been found as the outcome of this pandemic disease. Additionally, at the early stages of infection, COVID‐19 patients show dysmorphia and nausea, headache, vomiting, and sometimes cerebral damage in serious conditions when it spread to the brain.^39^, ^40^ SARS‐CoV‐2 virus can enter the brain through the cerebral circulation as the ACE2 receptor is present in the endothelium and cribriform plate near the olfactory bulb. Additionally, the potential targets in the brain are the ACE2‐expressing glial cells and neurons.^41^ This virus has a higher neuroinvasive ability than others for the expression of ACE2 in the brain on the surface of endothelial cells. This neurotropic virus causes the disease in the nerve endings first and then it slowly transmits to the brain via specific routes. Thus, it would directly damage the CNS by producing many proinflammatory cytokines.

### 2.1 Passage of SARS‐CoV‐2 across the BBB

The BBB is the major hurdle in the CNS for maintaining homeostasis and for the protection of the brain against pathogens. BBB permeability is increased by inflammation and vascular damage to potentiate unwanted CNS effects.^42^ SARS‐CoV‐2 can destroy the integrity of the BBB leading to secondary infections causing symptoms like headache, vomiting, loss of vision, and limb convulsions. Transcellular, paracellular, and retrograde axonal transport along the sensory and olfactory nerves are several ways for viruses to cross the BBB.^43^ Neurotropic properties of coronavirus are shown in several hosts including humans. The interaction of the S glycoprotein with the ACE2 on the endothelial cells provides slow vascularization in the capillary. The host cell invasion occurs via transcellular migration of the virus particles to overcome the resistance due to the presence of BBB. Furthermore, the tight junction in the BBB is attacked by the virus particles through paracellular migration. Additionally, axonal transport of viral particles can also occur via adherence to proteins of the peripheral or cranial nerves to allow reverse transport. In addition, axonal transport of the virus to the brain is provided by the cribriform plate beside the olfactory bulb that results in loss of smell in COVID‐19 patients.^44^, ^45^ Additionally, SARS‐CoV‐2 infection can cause migration of viral particles to reach cerebral circulation through the systematic spread. Moreover, the virus binds to ACE2 in the brain endothelial and smooth muscle cells. It also affects the favorable balance of the harmful ‘ACE2 ‐ angiotensin II axis that promotes neuronal tissue injury.^46^

## 3 NEUROLOGICAL BRAIN INJURY IN COVID‐19 – A DETAILED MECHANISM

Reported symptoms of SARS‐COV‐2 infection are dry cough with mild fever followed by shortness of breath, fatigue, dyspnea, and hypoxemia (low oxygen saturation level).^47^ Late stages of this disease lead to lymphopenia, increased LDH (lactate dehydrogenase), prolonged prothrombin time (PT), cellular immune deficiency, endotheliitis in vital organs, tissues, and ultimate organ injuries. In most cases, hyper‐coagulation, oxidative stress, and infiltration of macrophages in multiple organs are the worst scenarios. In a recent cohort study with 2,36,379 COVID‐19 patients, the incidence of neurological damage was 33.62% and the psychiatric occurrence was around 12·84% within 6 months of diagnosis. The individual diagnosis reported 46.42% neurological damage, 0.56% intracranial hemorrhage, 2.10% ischemic stroke, 0.11% parkinsonism, 17.39% anxiety disorder, 1.40% psychotic disorder, and 0.67% dementia in ITU (Intensive Therapy Unit) patients.^48^ Thus, it would be interesting to establish the possible neurological connections with the COVID‐19.

### 3.1 Cerebrovascular injury

Multiple mechanisms have been involved to increase the risk of ischemic stroke in COVID‐19 patients. The hypercoagulable state, increased embolic events, microangiopathic thrombosis, and endotheliopathy are all major events for ischemic stroke in SARS‐CoV‐2 infected patients.^49^ Viral infections in the cerebrovascular endothelium cause vasculitis in the brain region.^50^ Furthermore, thrombotic microangiopathy occurs due to local damage in the cerebrovascular system. ACE2 receptor homeostasis disruption in the cerebrovascular system alters angiotensin level, which impairs cerebral autoregulation. Additionally, increased HIF1 and HIF2 levels promote the coagulation cascade via activating the extrinsic coagulation pathway and inhibition of fibrinolysis with an elevated level of plasminogen activator inhibitor 1.^51^, ^52^

The virus and the endothelial ACE2 receptor interaction leads to increase luminal pressure in the vessels that results in rupture of the vessel wall and bleeding in the brain. Additionally, several abnormalities in the coagulation system such as thrombocytopenia, and intracerebral bleeding in COVID‐19 patients are due to increased D‐dimer formation.^53^, ^54^ These abnormalities have a certain impact on endothelial injury, venous stasis, and hypercoagulable states which ultimately promotes thrombosis. Furthermore, ACE2 dysregulation in infected patients results in post‐ischemic inflammation leading to perfusion in the ischemic zone and occasionally developing larger infarct volume.^55^, ^56^ Additionally, ACE2 dysfunctions impair cerebrovascular endothelium and contributes to pathogenesis and intracellular hemorrhage.^57^, ^58^ Cerebral venous thrombosis may also occur due to in situ thrombosis and hypercoagulopathy. SARS‐COV‐2 infection causes endotheliitis and vasculitis of the CNS leading to neuronal injury and ischemia or intracerebral hemorrhage.^59^ Bleeding disorder, prolonged PT and homeostasis dysfunction also occur in severely affected patients. In COVID‐19 patients, coagulopathy is associated with elevated D‐dimer levels but only mild thrombocytopenia and slightly prolonged PT take place.^60^

Serum angiotensin II level is also increased by the interaction of trans‐membrane serine protease 2 (TMPRSS2) with ACE2 receptors, resulting in a massive release of inflammatory cytokines together with monocyte‐derived macrophages and ultimately starts extrinsic coagulation pathway that results in fibrin elevation and blood clotting in ischemic stroke.^61^, ^62^ SARS‐CoV‐2 infection also disrupts the BBB by destabilizing tight junction proteins which can also cause an intracerebral hemorrhage. The increased serum levels of angiotensin 2 and decreased cerebral renin‐angiotensin system cause cerebral vascular dysregulation and impair endothelial function, which deregulates blood pressure and contributes to hypertension and hemorrhagic stroke. Additionally, endothelial dysfunction processes also lead to plaque rupture and thrombosis that results in the stroke where inflammatory markers are independent factors.^63^, ^64^ Crebrovascular problems in COVID‐19 patients can cause hypertension, diabetes, hyperlipidemia, and stroke. The risk factors associated with changed proinflammatory conditions are leukocyte activation and cerebrovascular thrombosis. Additionally, accumulated inflammatory cells in the vascular wall increase the permeability of BBB.^65^ Thus brain cell destruction and atherosclerotic plaque formation take place in the anterior and internal cerebral arteries.^66^

### 3.2 Hypoxic brain injury

In hypoxia, decreased oxygen supply to the tissues results in mitochondrial dysfunction, sepsis, intravascular coagulopathy, increased level of unbound haem due to hemolysis, low‐level of nitric oxide (NO) in the blood causes cytopathic hypoxia and ischemia in most of the SARS‐CoV‐2 patients. Hypoxia‐inducible factor‐1α (HIF‐1α) is ubiquitinated and degraded by prolyl‐hydroxylase domain proteins (PHDs) in normoxia whereas the hypoxic condition impairs the function of PHD and stabilizes HIF‐1α which in turn stimulates vascular endothelial growth factor (VEGF), tumor necrosis factor (TNF), IL‐1 and IL‐12 secretion.^67^ It also induces glycolytic enzymes (e.g., hexokinase, pyruvate dehydrogenase kinase1, and pyruvate kinase isozymes M2) as well as glucose transferases which helps to alter energy production cycles in dendritic cells (DCs), neutrophils and macrophages. HIF‐1α is also a major deciding factor for M1 macrophage polarization and activation of IL‐1β, TNF‐α, signal transducer and activator of transcription 3 (STAT3), inducible NO synthase (iNOS), and IL‐23. In SARS‐CoV‐2 infection, hypoxia, and Toll‐like receptor 4 (TLR4) induce HIF‐1α in macrophage that leads to an increase in A Disintegrin and Metalloproteinase 17 and decreases ACE2 and TMPRSS2 levels.^68^, ^69^

Thus, systemic hypoxia in COVID‐19 patients is associated with cytokine release, involvement of TNF‐α, and other inflammatory factors and reduces tO_2_ levels.^70^, ^71^ Moreover, the cellular oxygen sensors activate the HIF which triggers cellular adaptation to hypoxia which ultimately elicits inflammatory responses.^72^, ^73^ High cytokine levels and/or reduced brain tO2 can affect cognitive functions and quality of life in infected patients associated with hypoxia where brain serotonin level is diminished.^74^

As a major concern in COVID‐19 pathophysiology, generally, two types of events occur during a hypoxic situation. The early event is the damage of pneumocytes in the lung alveoli and this can cause alveolar damage to up to 10% of lung volume.^75^, ^76^ The second cause is due to induced hypoxia for neuroinvasive damage in CNS.^77^, ^78^ In addition to all these facts coronaviruses may also enter the brain through the trans‐synaptic route and reach in medullary cardio receptor center from the lungs and lower respiratory airways. That's why anosmia and ageusia occur in COVID‐19 patients.^79^, ^80^ Old age is also critical for death in COVID‐19 due to the diminished nature of respiratory responses to hypoxemia and hypercapnia.^81^ Additionally, cerebrovascular and ventilatory activity to hypoxia of older patients are also reduced.^82^

### 3.3 Mechanism of neurological damages

The intricate mechanistic disturbances of neurological functions due to SARS‐CoV‐2 infection is mostly unknown. The genome sequence of SARS‐CoV‐2 along with the occurrence of cerebral edema and meningeal vasodilation has also been found in autopsy studies of the brain in several COVID‐19 patients as this virus causes different neurological diseases including ischemic stroke.^83^, ^84^ SARS‐CoV‐2 is 79.5% similar in genetic consequences with SARS‐CoV and 50% to Middle East respiratory syndrome coronavirus (MERS‐CoV) shows neurological symptoms, cerebral hemorrhage, and infarction without showing its typical symptoms.^85^, ^86^ Furthermore, abnormal blood clotting in arteries has happened in COVID‐19 that results in severe stroke.^87^, ^88^ Additionally, high D‐dimer formation and reduced platelet levels were shown in patients vulnerable to acute cerebrovascular dysfunction. Alveolar gas exchange in the lung tissues may be impaired and triggered CNS hypoxia leads to anaerobic metabolism in brain cells with acid accumulation that causes cerebral vasodilation, brain cell swelling, interstitial edema, cerebral blood flow, and headache due to ischemic congestion. Hypoxia develops due to low levels of oxygen in the blood and damages the brain and other vital organs. In high‐risk COVID‐19 patients, the acute cerebrovascular disease may be induced by untreated hypoxia. Thus, COVID‐19 patients with several neurological and cerebrovascular dysfunctions like ischemic stroke‐affected brain micro‐capillaries. Older patients with SARS‐CoV‐2 infection have hypercoagulable blood due to increased inflammatory responses.^49^, ^89^ Hypercoagulability leads to microthrombi formation in the vessels of COVID‐19 patients. Furthermore, coagulation abnormalities trigger inflammatory factors like interleukins and C‐reactive proteins (CRPs), which are responsible for the initial molecular events. ACE2 facilitates penetration of SARS–CoV‐2 in the host cells with the transformations of angiotensin I to angiotensin II, which have vasodilatory and antifibrotic effects in COVID‐19 patients. Mao *et al*, 2020 showed that autopsy results indicate that the COVID‐19 patient's brain tissue was hyperemic, edematous, and presence of some degenerated neurons.^18^ Pathophysiological effect of SARS–CoV‐2 infection leads to prolonged hypoxia with neurological manifestations that involve acute intravascular events including disruption of blood supply. The good news is that reperfusion therapy can recover the damage created due to ischemic stroke in COVID‐19 patients.^90^ Hyper‐inflammatory response, hypercoagulable state, and coagulopathy are indirect factors whereas the interaction between ACE2 and virus initiates the direct events. There is increasing evidence of olfactory dysfunction in COVID‐19. Loss of smell and taste of the patients are now accepted as the fundamental symptoms of COVID‐19. Furthermore, SARS‐CoV‐2 infection causes neurological syndromes like anosmia to severe ischemic stroke or intracerebral hemorrhage.^91^, ^92^ Additionally, cytokine storm leads to the hypercoagulable state and vascular thrombosis in COVID‐19.^93^ So, there might be a direct association between COVID‐19 and neuronal damage in the brain causing cerebral ischemia.

ACE2 expression is seen in the olfactory bulb, neurons, astrocytes, and oligodendrocytes. The virus can spread in important brain areas when the olfactory epithelium is infected, a main passage of SARS‐COV‐2 through the brainstem which is operating mainly to control blood pressure, heartbeat, and respiration. Cerebral vascular endothelial is also infected by SARS‐CoV‐2 via infected leukocytes and then it moves to the CNS.^94^ Respiratory failure may be occurred due to pulmonary lesions or brainstem infection. Thus, in COVID‐19 the intracranial infection leads to epilepsy, confusion, and headache. Moreover, these features generally emerge from pulmonary infection resulting in dyspnea, cough, and fever. Pneumonia is also presumed to be responsible for cerebral stroke in COVID‐19.^95^, ^96^

Another case study of a patient with virus‐associated cerebral infarcts by brain imaging showed increased white blood cell counts and clotting modules including an elevated PT, increased D‐Dimer and fibrinogen levels, markedly increase ferritin level, and anticardiolipin antibody.^97^ Additionally, non‐specific hyperferritinemia, a marker of inflammatory response is observed in antiphospholipid syndrome and its variance (catastrophic antiphospholipid syndrome) that leads to arterial and venous thrombosis. In COVID‐19 patients, the inflammation flows from one region to another in the brain through white matter containing long nerve fibers and wires due to lack of oxygen or injury and leaky blood vessels. Additionally, in these patients’ cognitive difficulties are observed due to diffused white matter. Increased anticardiolipin immunoglobulin M antibodies and antiphospholipid syndrome have also been observed in cerebral ischemic patients with SARS‐CoV‐2 infection and hyperferritinemia. The increased binding of the spike protein of SARS‐CoV‐2 to ACE2 is found both in the epithelial cells and vascular endothelium.^98^, ^99^, ^100^ That's why COVID‐19 patients are on the verge of risk of cerebral ischemia and thrombogenesis due to the presence of both a hypercoagulable state and vascular injury.

## 4 REDOX IMBALANCE IN COVID‐19

In COVID‐19, cytokine storm induces ROS, the major causing agent for all these histological, inflammatory, and cellular changes in the infected hosts. ROS play a major role in many diseases including ischemia‐reperfusion injury and stroke due to the excess production of reactive oxidants, decreased antioxidant levels, and NO bioavailability in the vasculature.^101^, ^102^ ROS production as intermediates in redox reactions comprised of two major groups: highly reactive free radicals containing unpaired electrons, for example, superoxide, hydroxyl ion, NO, and non‐radicals, for example, H_2_O_2_ and ONOO^−^ with less reactivity with a longer half‐life.^103^, ^104^

### 4.1 Generation of ROS

The primary source of ROS production by several biochemical reactions inside the mammalian cells is the mitochondrial electron transport chain (ETC). The sequential flow of electrons through ETC produces ATP and H_2_O. But oxidative stress and TNF‐α produce ROS by using leaky electrons in the ETC complex I, III, and glycerol 3‐phosphate dehydrogenase.^105^ Cytoplasmic NADPH oxidase (NOX) family proteins also produce a large amount of ROS which is regulated by transforming growth factor beta (TGF‐β) signaling. Xanthine oxidase in a hypoxic condition also produces a huge amount of ROS by the Haber‐Weiss‐Fenton reaction. Additionally, the cytochrome P450 monooxygenase system generates different ROS species by abnormal uncoupling of xenobiotic metabolism pathways.^106^, ^107^, ^108^

### 4.2 Mode‐of‐action of ROS

**FIGURE 2:**
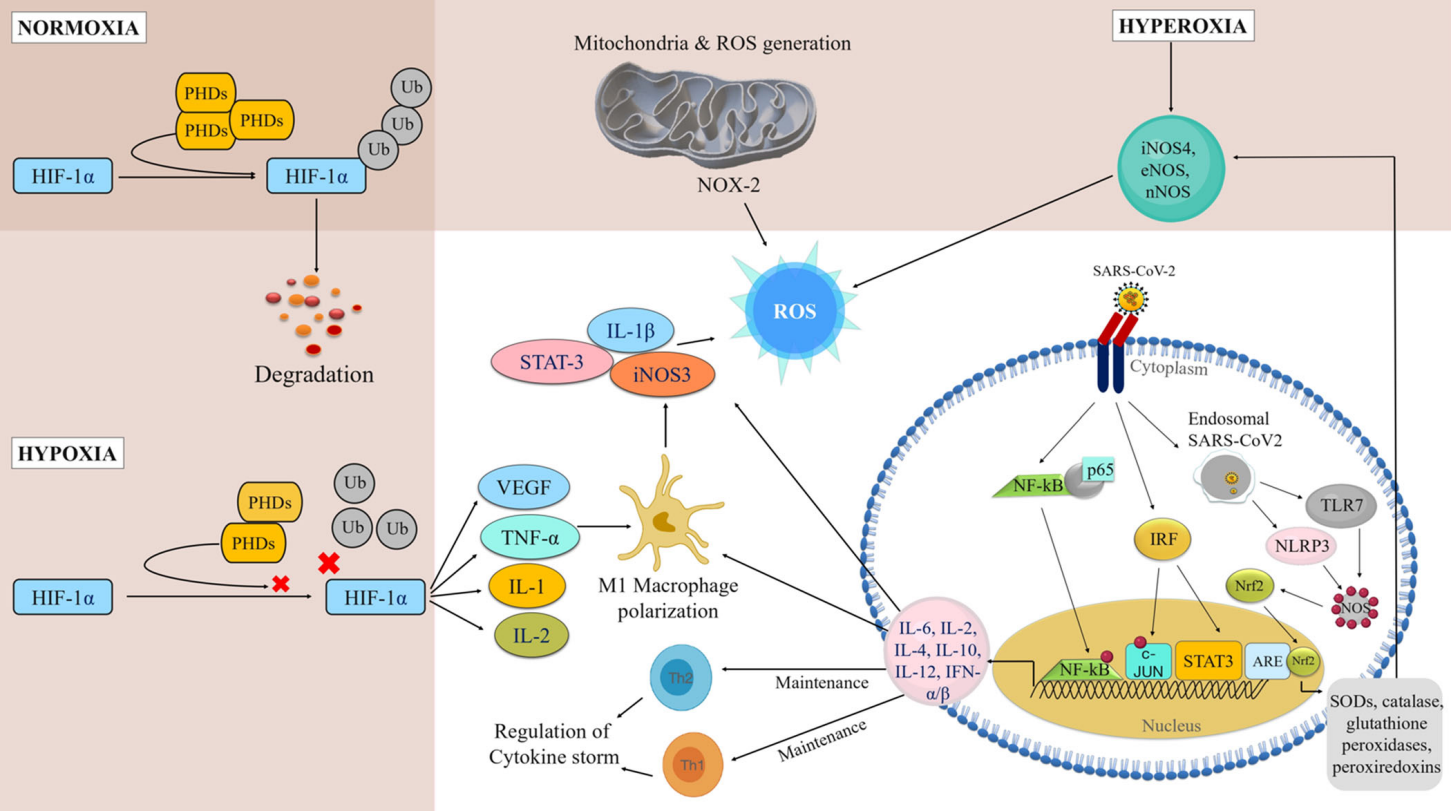
Physiological context of reactive oxygen species (ROS) generation in hypoxic and hyperoxic conditions that are connected with the major immune signaling cascades to participate in coronavirus disease 2019 (COVID‐19) severity (*use of Biorender as tools to generate the figure*).

Recent studies reported that some viruses alter redox balance in host cells to facilitate their own replication by manipulating host nuclear factor E2‐related factor 2 (Nrf2) for their propagation. They induce lipid peroxidation to reduce the expressions of Nrf2 target genes hemoxigenase‐1, superoxide dismutase (SOD), glutathione S‐transferase, glutathione peroxidase (GPx), and catalase to control the redox balance.^109^ Altered redox balance increases by viral pathogenesis due to the induction of oxidative stress and causes cell death. Host's nonspecific immune defense system initiated by pro‐inflammatory cytokines, interferon (IFN), TLRs, and nuclear factor kappa‐light‐chain‐enhancer of activated B cells (NF‐κB) pathways also induce oxidative stress.^110^ During inflammation in COVID‐19, the epithelial cells express cytokines, chemokines, and growth factors. Furthermore, the interleukin family also recruits leukocytes to the sites of infection.^111^ IL‐6 expression is significantly higher that mediates acute phase immunological responses resulting in increased body temperature as well as activation of inflammatory immune cells predominantly neutrophils and macrophages. In infected cells, cytokines upregulate ROS activity and elevated ROS‐induced expressions of cytokines in a cyclic fashion like a positive feedback loop mechanism. As a result, in COVID‐19 patients, extensive and life‐threatening parenchymal tissue damage occurs (Figure 2).^112^, ^113^

### 4.3 Redox imbalance

Renin‐Angiotensin‐Aldosterone System (RAAS) plays a major role to maintain redox balance. It is composed of two sub‐axes *viz*. antioxidant axis (ACE 2 and Angiotensin 1–7 (Ang1‐7)/Mas receptor) and pro‐oxidant axis (ACE & Ang II/AT1).^114^, ^115^ In this system, renin cleaves angiotensin to form angiotensin I which is subsequently chopped by ACE2 into angiotensin II. Furthermore, ACE2 cleaves Angiotensin II into Ang 1–7. ACE2 activity decreases after SARS‐CoV‐2 binding. That is why angiotensin II level increased, and it binds with the angiotensin II type 1 (AT1) receptor that triggers Mitogen‐activated protein kinase (MAPK), Protein kinase C, and NF‐κB pathways as well as activates endothelial NOX2 and cyclooxygenase 2.^116^ ROS produced by endothelial NOX2 sequentially upregulates mitochondrial ROS (mtROS) production. Furthermore, aldosterone induces oxidative stress, hypertrophy, fibrosis, and inflammation.^113^ In SARS‐CoV‐2 infection, downregulation of the ACE2/Ang/Mas axis and hyperactivation of the Ang II/AT1R pathway II causes NOX/ROS signaling pathway activation.^117^ Ang II, induced by NOX‐derived ROS, promotes activation of the TGFβ1‐Smad2/3 signaling pathway. Additionally, oxidative stress induces ACE2 hypo‐methylation which leads to the upregulation of ACE2 and facilitates SARS‐CoV‐2 receptor binding.^118^

## 5 IMMUNE CASCADE DYSREGULATION LEADS TO SUPPRESSED IMMUNITY

Different ROS follows different routes of signaling pathways leading to divergent and potentially opposing functional responses. The main signaling target for both SARS‐CoV‐2 and proinflammatory cytokines is endothelium and its surface adhesion molecules (e.g., vascular cell adhesion protein 1, intercellular adhesion molecule 1, and E‐selectin).^119^, ^120^

### 5.1 Inflammatory immune responses

**FIGURE 3:**
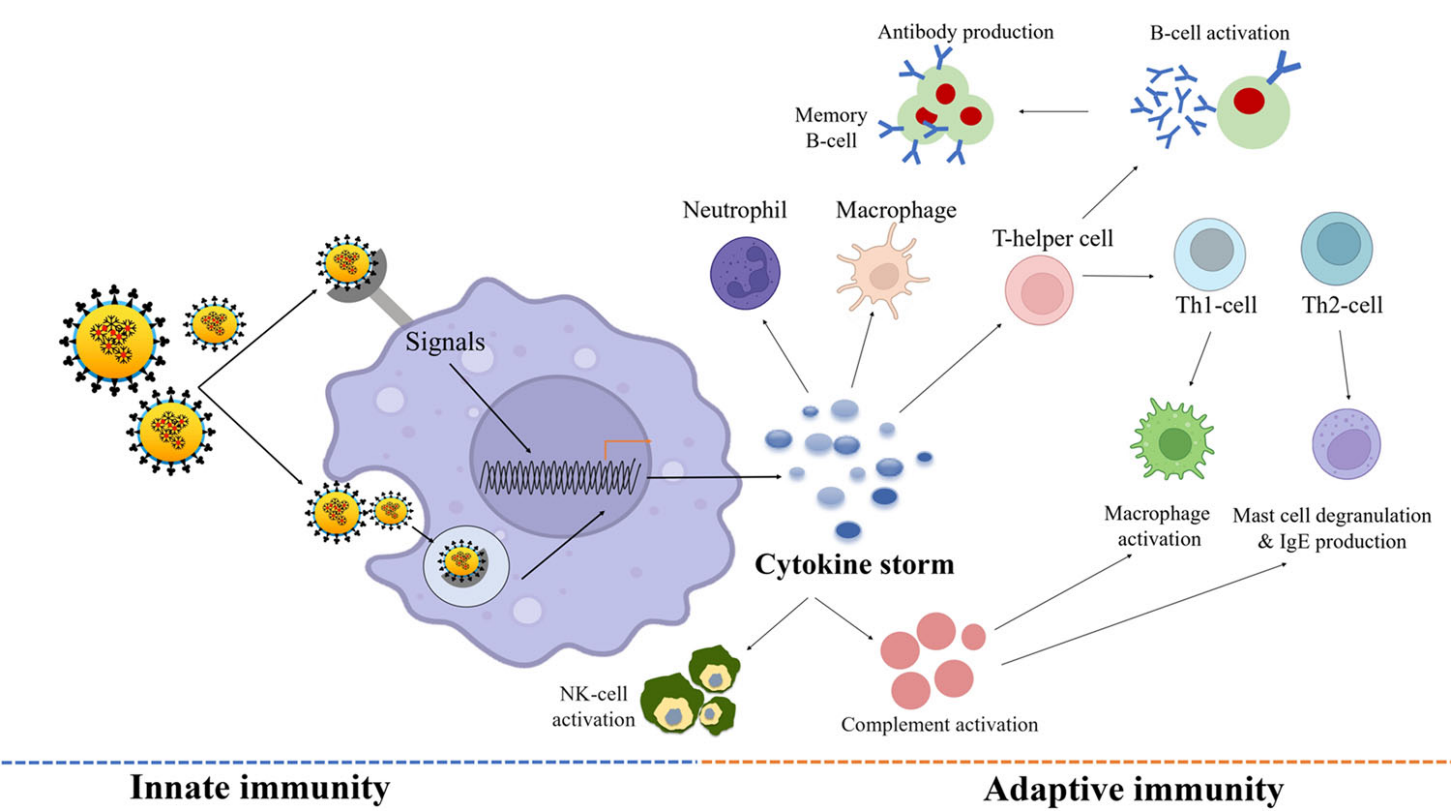
Severe acute respiratory syndrome coronavirus 2 (SARS‐CoV‐2) infection affects the bridge between the innate and adaptive immune systems (*use of Biorender as tools to generate the figure*).

Following the initial infection of lung alveolar epithelial cells, the immune system gets triggered through the inflammatory route. After entry, the virus is engulfed by antigen‐presenting cells (APCs), macrophages, and DCs, and processed antigens to T‐cells that lead to their activation, differentiation, and secretion of cytokines. Viral infection is immediately recognized by the host's innate immune system using pattern recognition receptors (PRRs) and TLRs for recognizing virus‐pathogen‐associated molecular patterns (e.g., viral proteins, lipids, and nucleic acids). Thereafter, different pathways like NF‐κβ, interferon regulatory transcription factor, and MAPK are activated which results in the induction of expressions of the inflammatory cytokines (e.g., IFN, IL‐1β, IL‐6, etc.). The large‐scale uncontrolled cytokine storm generally presents itself in the form of systemic inflammation. IL‐6 and IL‐1β are the predominant cytokines released during the early stage of viral infections.^121^ IL‐1β in particular is responsible for promoting inflammation in the alveoli and bronchi of patients exhibiting pulmonary injury. On the other hand, higher levels of IL‐6 can stimulate the complement system which greatly enhances the vascular permeability, thereby giving the circumstances for the further spread of the infection. At the same time, anti‐inflammatory factors such as type I interferons and IL‐10 were generally found to be depleted during such incidences of SARS‐CoV‐2 infection (Figure 3).^122^

It has been clinically observed that severe patients have an induced cytokine profile induced by SARS‐CoV‐1 and MERS‐CoV.^123^, ^124^ CRP and erythematous sedimentation rate were also found to be elevated in severe COVID‐19 patients. These symptoms were related to hyper‐coagulation, acute respiratory distress syndrome (ARDS), and disseminated intravascular coagulation, and presented in thrombosis, thrombocytopenia, and gangrene on the limbs.

### 5.2 Dysregulation of immune signatures

**FIGURE 4:**
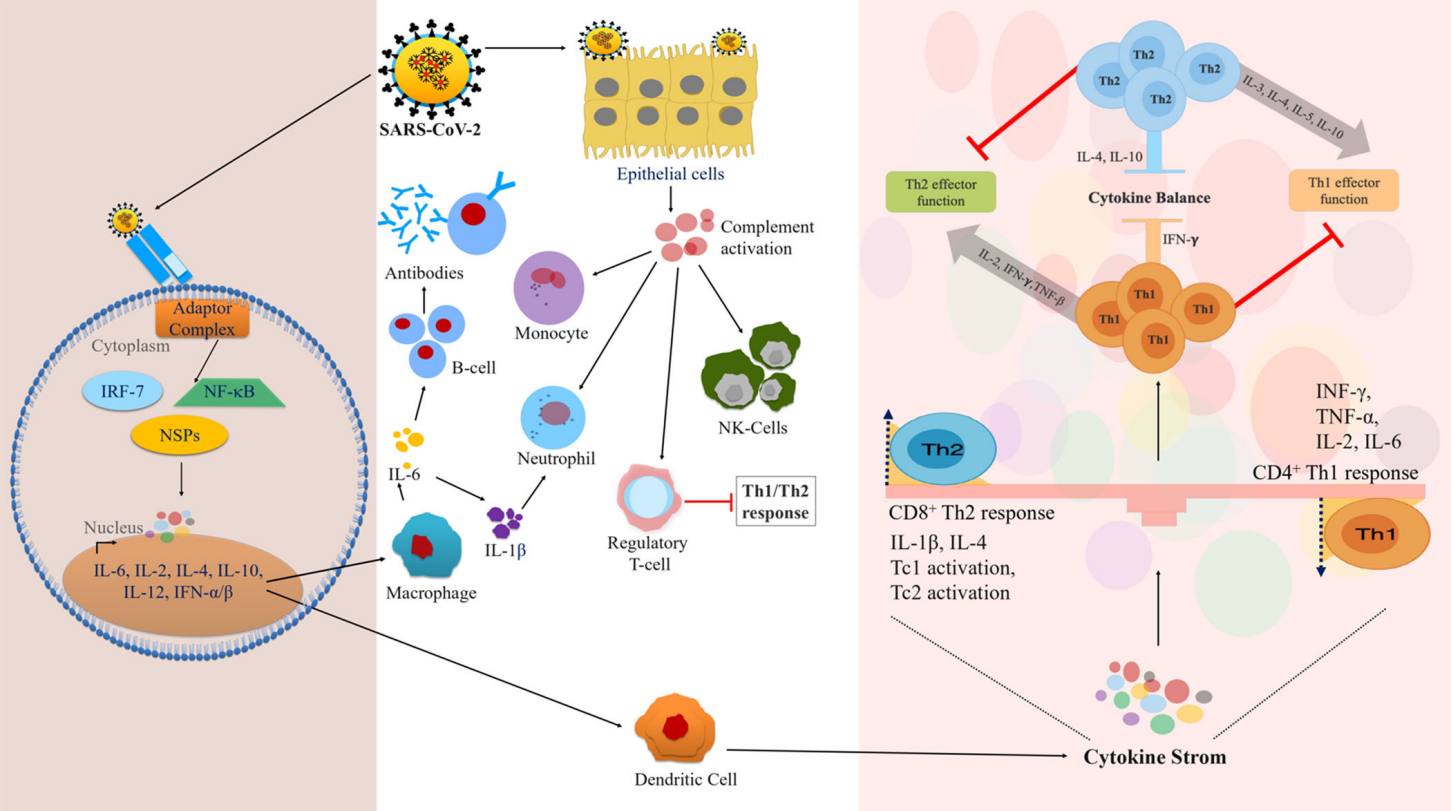
Severe acute respiratory syndrome coronavirus 2 (SARS‐CoV‐2) mediated dysregulation of immunological machinery. SARS‐CoV‐2 binds to the receptors that lead to cytokine storm which affects the Th1 and Th2 mediated homeostasis.

Expression of cytokines increased by PRR, for example, TLR 3, 7, and 8, Nucleotide Binding Oligomerisation Domain (NOD)‐like receptor family members and retinoic acid‐inducible gene‐1 in macrophages, DCs, and lung epithelial cells.^125^, ^126^ Elevated ROS is the major mediator for NOD‐like receptors P3 (NLRP3) and involves in NF‐κB activation. Increased levels of IL‐6, IL8, IL‐1β, TNFα, CXCL10, macrophage inflammatory proteins 1α (MIP‐1α), granulocyte CSF, and C‐C motif chemokine ligand 2 (CCL2/ MCP‐1) are also reported in COVID‐19 (Figure 4).^127^, ^128^ Activated neutrophils and macrophages produce a huge amount of ROS leading to respiratory bursts under oxidative stress. ROS along with free heme from dense matted deposits by converting soluble plasma fibrinogen into abnormal fibrin clots leads to micro‐thrombosis in the vascular system which further causes ischemia in pulmonary micro‐circulation.^10^, ^129^

Multiple isoforms of NOX and xanthine oxidase are oxidative stress‐inducing enzymes to generate hydrogen peroxide by the dismutation of superoxide anion. They are the major sources of ROS production in immune cells during phagocytosis.^130^, ^131^ In COVID‐19, SARS‐CoV‐2 infection is associated with endosomal NOX activation by TLR7. In a normal situation, proteasomal degradation of nuclear factor erythroid 2‐related factor‐2 (Nrf2) is mediated by Kelch‐like ECH‐associated protein 1 (KEAP1). TLR7‐NOXs help Nrf2 nuclear translocation through the inactivation of KEAP1 for ROS production.^132^, ^133^, ^134^ Furthermore, Nrf2 attaches to antioxidant response elements and upregulates antioxidants, for example, catalase, superoxide dismutase, GPxs, and peroxiredoxins.

### 5.3 Suppression of immunological machinery

The entire immune system of COVID‐19 patients collectively promotes the uncontrolled production of cytokines and chemokines known as a cytokine storm.^135^, ^136^ During its preliminary stage, SARS‐CoV‐2 activates the dendritic and epithelial cells to trigger the release of numerous proinflammatory cytokines and chemokines, for example, IL‐1β, IL‐2, IL‐6, IL‐8, IFN‐α/β, TNF, CXXC, CCL3, CCL5, CCL2, IP10, and so forth.^137^, ^138^, ^139^, ^140^ Several studies also suggest that both innate and adaptive immune systems are responsible for inflammatory immune responses. It is likely that the innate immune system responded against SARS‐CoV‐2 infection by producing some proinflammatory cytokines as well as through the secretion of chemokines. Here in this section, we are trying toexplain how SARS‐CoV‐2 mediates immunomodulation through an interplay with innate and adaptive immune systems. Finally, how dysregulation of immune cells by SARS‐CoV‐2 leads to the occurrence of neurological disturbances.

A highly variable and diverse immune system has been developed in an infected host. Invading pathogens and the body's own cells can be distinguished by its complex network of cells and molecules. Unlike other acute infections, a long‐lasting immunological memory does not persist by SARS‐CoV‐2 but rather progresses.^141^ It also requires repeated infections to continue. The progressive immunity is not constant as viral particles or viral titer will go remarkably high after some days of the infection and the survival chance of the host will go down. However, reverse conditions may lead to low levels of the viral load so that severe clinical symptoms do not observe in some COVID‐19 patients. Semi‐immunity development is induced by age, genetic background, pregnancy, co‐infections, and the nutritional status of the patient. It has been reported that in COVID‐19 patients, the number of white blood cells, neutrophils as well as levels of procalcitonin, CRP, and other inflammatory indices are remarkably high in the intensive care unit (ICU) cases than in non‐ICU patients.^142^, ^143^ Most studies presented pro‐inflammatory cytokines; especially IL‐6 concentrations are at higher levels in severely affected individuals compared to mild COVID‐19 patients. Additionally, the existence of ARDS and T‐cell overactivation as well as immunosuppression of lung macrophages are shown from the postmortem examination of those who died from COVID‐19 due to high lung infection.^144^, ^145^, ^146^

Uncontrolled inflammatory immune responses have been developed by the SARS‐CoV‐2 infection as it takes part in both the innate and adaptive immune systems. An early IFN‐γ response to COVID‐19 is also important in protecting the evolution of severe disease symptoms.^147^, ^148^ Cytokine storm generation by stimulating pathogens can also start the adaptive immune responses. Additionally, PRR mediates the stimulation of the innate immune system. It also binds to the conserved molecular structures found in large groups of pathogens.^149^ Additionally, natural killer (NK) cells have been involved as a source of early pro‐inflammatory reactions through IFN‐γ and TNF‐α in some respiratory diseases. But in other studies, it has been pointed to γδΤ cells and αβT cells.^150^ Another report from lethal SARS‐CoV‐2 infection leads to excessive cytokines and chemokines production and ultimately involves the APCs like macrophages and T cells. It was also reported that virus titers in COVID‐19 patients are significantly higher in the case of TLR3 that recruits a high amount of adapter‐inducing interferon‐b (TRIF) in the TIR‐domain of the adaptor molecules leading to high IL‐6 and IL‐1beta production in immunocompromised mice in comparing to their wild‐type counterparts leading to severe lung damage.^151^

In addition, in the innate immune responses of SARS‐CoV‐2 infection, adaptive immunity acquires CD4^+^ T‐cells which are becoming the major producers of cytokines.^152^ In the early stimulation phase, IL‐2 is produced by Helper T (Th) cells, designated as Th0. The Th cells are polarized towards either Th1 or Th2 subsets depending on the nature of the cytokines present at the site of activation as the antigen stimulation continues.^153^ INF‐γ, TNF‐α, IL‐2, IL‐6 and IL‐9 are produced by CD4^+^ Th1 cells. Collectively, SARS and Middle East Respiratory Syndrome (MERS) have certain similarities in case of their pathological, clinical, and immunological features. SARS‐CoV‐2 shows a prolonged lung infection with elevated levels of NKG4 (exhaustion marker) promoted by NK cells followed by CD8^+^ T cells.^85^, ^154^ Non‐structural proteins, nsp9 and nsp10 of SARS‐CoV‐2 targets NKRF (NF‐kB repressor).^155^ IL‐6 and IL‐8 production is also promoted in immunocompromised individuals which were established in a study on peripheral blood mononuclear cells (PBMCs) assessment. Additionally, patients with COVID‐19 express human leukocyte antigens (HLA) haplotypes which are capable of inducing CD8^+^ T cell‐mediated responses.^156^

The neuroprotective function of T‐reg cells leads to the production of IL‐10, an anti‐inflammatory cytokine that targets more than 300 inflammatory pathway genes.^157^, ^158^ A well‐known target is TGF‐β which has a neuroprotective function in post‐ischemic injury by directing protective effects on the cells during ischemic stroke.^159^, ^160^ Therefore, post‐ischemic involvement of IL‐10 and TGF‐β is necessary to bridge the gap between the innate and adaptive immune systems during ischemic injury.

## 6 CROSSTALK BETWEEN ROS AND IMMUNE CASCADE

Activated endothelial cells express surface adhesion molecules and produce chemokines and proinflammatory cytokines that result in leucocyte migration and recruitment to the infected areas. This niche amplifies the immune response and inhibits the sphingosine‐1‐phosphate pathway which leads to the initiation of cytokine storm and lung edema. ROS activates TNF and NF‐κB for regulation of the expression of GTPases (Rho and Rac1), and matrix metalloproteinases, which in turn activate an array of adhesion molecules and proinflammatory cytokines (e.g., IL‐1, IL‐6, IL‐8, TGFβ1, and TNF).^161^, ^162^ mtROS along with NOXs control expression of the Receptor for Advanced Glycation End Products, TNF, and NF‐κB which in turn regulate caspases and expressions of IL‐1β and IL‐6. It is also reported that IL‐6 induces mtROS production by activating NOX and involves activating STAT3, MAPK, and Notch pathways. Furthermore, MCP1 expression is upregulated by STAT3 for neutrophil and monocyte activation.^163^, ^164^, ^165^ Furthermore, the “IL‐6 ‐ STAT3” axis downregulates endothelial NOS which results in vasomotor endothelial dysfunction due to low levels of NO. Additionally, in oxidative stress, VEGF is upregulated by angiotensin II and IL‐6 and plays important role in angiogenesis in the SARS‐CoV‐2 infected lesions. Endothelial cells, type II alveolar epithelial cells, and non‐immune cells produce ROS via mitochondrial ETC.^166^ SARS‐CoV‐2 infect type II pneumocytes and impair their mitochondrial cycles, which in turn over‐activates the immune responses that felicitate viral replication. Additionally, the Nucleotide‐binding domain, leucine‐rich containing family, Pyrine domain containing 3 (NLRP3) inflammasome, IL‐1β, and mtROS production is regulated by SARS‐CoV‐2 non‐structural protein 3a.^167^

Both ROS and RNS play major roles in cerebral ischemia. ROS and RNS on the one hand act as vital signaling molecules in immune cells, on the other hand, cause cell signaling alteration and tissue damage. A low level of ROS production by alveolar macrophages helps in immune defense, and virus phagocytosis, and maintains cellular signaling pathways. But the expression of NO synthase 2 (NOS2) increases the level of ROS which initiates the synthesis of RNS and induces nitrosative stress inside the tissues in addition to ROS‐mediated stress.^168^

## 7 NEUROLOGICAL DAMAGES AND CONSEQUENCES OF COVID‐19

SARS‐CoV‐2 is neuroinvasive and neurovirulent in nature and responsible for different neurological diseases including ischemic stroke. Another coronavirus MERS CoV causes MERS (Middle East Respiratory Syndrome) and is also neuroinvasive.^169^ Recent studies strongly indicate that COVID‐19 is not only restricted to pulmonary diseases but encompasses different neurologic manifestations like acute cerebral infarction.^170^ Comparative analysis of COVID‐19 patients with and without ischemic stroke indicates that severity is more in ischemic patients with SARS‐CoV‐2 infection.

### 7.1 Short and long‐run consequences

COVID‐19 occasionally acts to develop acute cerebrovascular diseases.^171^, ^172^ Mortality rates and worse outcomes are seen in COVID‐19 patients with stroke. Some viruses cause encephalitis and meningitis after entering the CNS. It may be due to the direct infection in vascular endothelium to invade olfactory epithelium through nasal cells or by migration with the leukocytes across the BBB and neuronal pathways to the Virchow‐robin space surrounded by arterioles and venules of the lymphatic system. Receptor attachment by the virus is generally found on certain blood vessel cells causing infection in arteries and veins, especially in the brain, and occasionally acts to develop acute cerebrovascular diseases.^173^, ^174^ Furthermore, abnormal blood clotting in arteries occurs in COVID‐19 patients that ultimately results in severe stroke.^175^, ^176^, ^177^

The major complications of COVID‐19 are ARDS, cardiac arrhythmia, acute cardiac injury, shock, pulmonary embolism, and cytokine release. The secondary infection leads to worse outcomes including cerebral stroke and increased mortality rates. Right limb weakness has been observed in a particular case of 60 years old hospitalized COVID‐19 patient.^178^ It has been reported that during treatment, the disease status was noticed after starting ischemic stroke although the patient has complications of frequent pneumonia after the stroke. Aging, oxidative stress, endothelial dysfunction, inflammation status, and other vascular risk factors play major roles in ischemic stroke with COVID‐19 infection.^95^, ^179^ Occasionally hypoxia and inflammation induced by SARS‐COV‐2 infection mostly provide the occurrence, development, and prognosis of stroke. Right limb weakness in COVID‐19 patients is initially due to hypoxemia and inflammatory cytokine secretion that occasionally results in acute ischemic stroke. As hypoxia continues, increased calcium ion concentration results in sequential cellular damage. Additionally, hypoxia may instigate inflammatory responses which include inflammatory cell infiltration and the release of cytokines that leads further to tissue or even organ‐level damage.^180^

### 7.2 Neurological damages in COVID‐19 leads to multi‐organ failure

**FIGURE 5:**
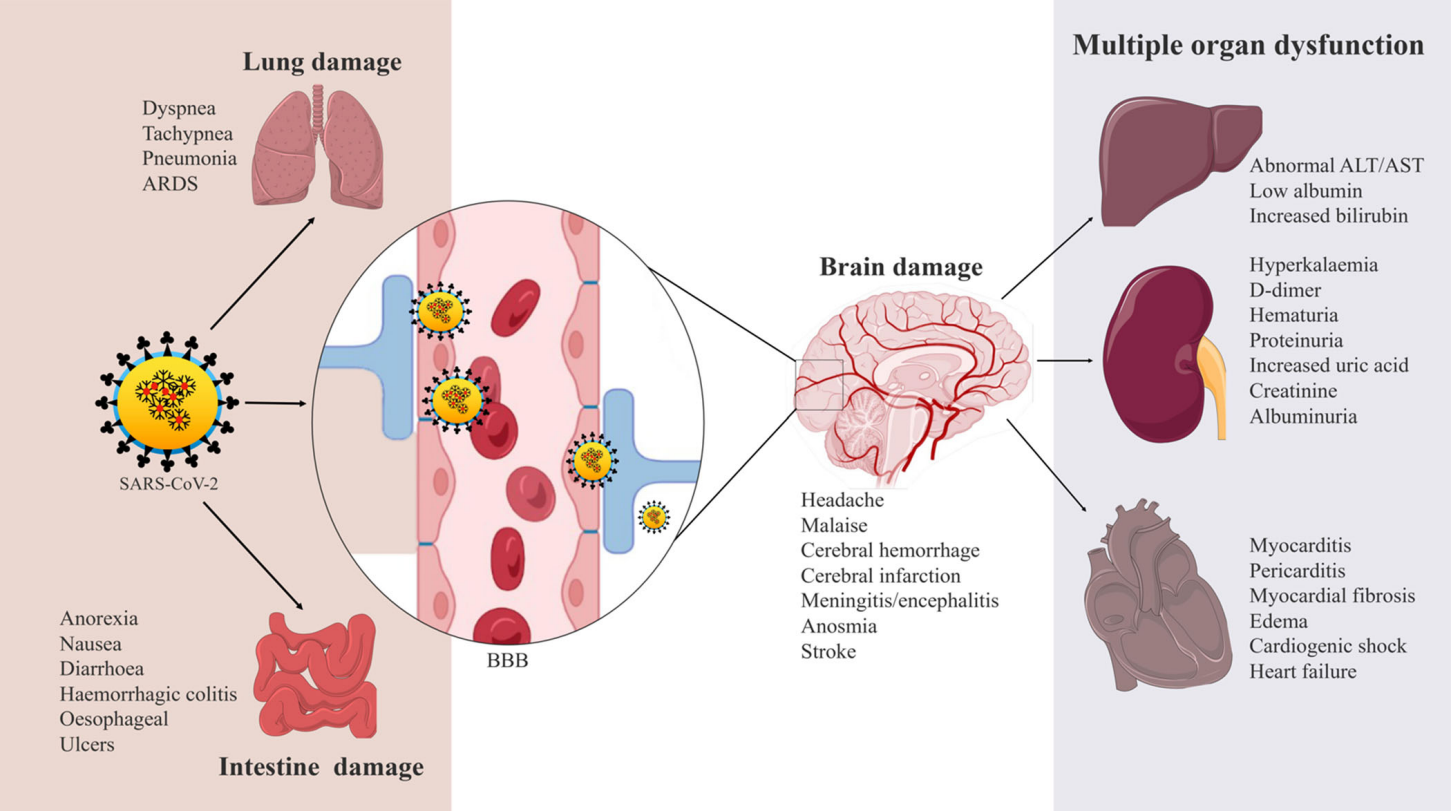
Organ‐specific manifestations in coronavirus disease 2019 (COVID‐19) due to the spreading of severe acute respiratory syndrome coronavirus 2 (SARS‐CoV‐2) infection in other organs and failure of neuronal control after brain damage (*use of Biorender as tools to generate the figure*).

Cytokine storm initiated by SARS‐CoV‐2 leads to severe acute cerebrovascular dysfunction and multiple organ failure (Figure 5). Activation of prothrombic pathways leads to cerebral stroke and affects the cerebral nervous system's parenchymal and vascular inflammatory response which dysregulates various neurological manifestations.^181^, ^182^ An intense inflammation and cerebral blood vessel injury also occur when the virus invades the endothelial cells. This cerebral nervous system invasion affects other organs such as the lungs, kidneys, heart, and liver.^183^ Meningio‐encephalitis, acute hemorrhagic necrotizing encephalopathy, and acute disseminated encephalomyelitis have also been observed in recent studies.^184^, ^185^ Cerebrovascular damage associated with other organs is at increased risk in COVID‐19. SARS‐CoV‐2 virus infects severely the respiratory and gastrointestinal tracts at an early stage.^135^, ^186^ Moreover, it has been observed that COVID‐19 is not only a respiratory pathogen, various neurological complications arise including confusion, stroke, and neuromuscular disorders. Even in young people, different symptoms have been observed that may last for a long time resulting in acute COVID‐19. Long COVID‐19 neuropsychiatric syndrome includes cognitive dysfunction, weakness, and headache in patients with severe illness along with respiratory and metabolic disturbances.^187^ It can also make rapid invasion to multiple organs, for example, lungs, heart, kidneys, bowel, liver, and brain causing some dysfunction syndrome.

Endothelial cells, seminal vesicles, gallbladder epithelium, renal proximal tubule, cardiomyocytes, testicular sertoli, and Leydig cells also express ACE2 receptors on their cell surfaces.^188^ Furthermore, ACE2 is expressed in the brush border of proximal cells of the kidney, which can also be injured due to SARS‐CoV‐2 infection resulting in proteinuria, hematuria, and elevated serum creatinine and blood urea levels. ACE2/Ang balance controls normal kidney functions but renal abnormalities and edema have also been observed in severely infected patients.^61^ Gastrointestinal system can also be dysfunctional as ACE2 is abundantly revealed in the intestinal luminal surface. Thus, these patients also appear symptoms like abdominal pain, nausea, diarrhea, and vomiting.^189^ From histopathology examination, mesenteric ischemia as well as diffuse endothelial inflammation has also been noticed although the gastrointestinal illness has not been directly related to death except for the long duration of gastrointestinal problems with elevated levels of alanine transaminase, aspartate transaminase, and total bilirubin.^190^, ^191^ COVID‐19 disease progression by ACE2 imbalance and RAAS activation can also lead to multiorgan dysfunction, especially in patients having diabetes mellitus, hypertension, and cardiovascular disorders.^192^ Besides this, excessive cytokine release, immune depression, and neurological dysfunction as a result of the cytopathic effect of the virus are the mechanisms for multiorgan dysfunction and ultimate failure in COVID‐19.^6^ Based on all these findings, preventive and therapeutic possibilities in COVID‐19 were highlighted below.

## 8 PREVENTIVE INTERVENTIONS FOR NEUROLOGICAL DAMAGES CAUSED BY SARS‐COV‐2

Inflammation plays a major role in SARS‐CoV‐2‐mediated lung injury and brain damage. Some inflammatory mediators in COVID‐19 are noted here (Table 1). Specific treatment for COVID‐19 is lacking though antiviral therapy, corticosteroid therapy, and mechanical respiratory support have been used.^193^ The viral pathogenesis and clinical features of the acute respiratory disorder have suggested that excessive inflammation, oxidative stress, and increased immune responses contribute to COVID‐19 pathology.

**TABLE 1:** Major function of antioxidants on chemokines, pro‐inflammatory and inflammatory cytokines.

**Chemokines /cytokines**	**Major functions in viral infection**	**Status**	**Ref**.
**Melatonin**			
IL‐8/CXCL8	Activate antimicrobial neutrophils and γδ T‐cells	⊥	^253^
MIG/CXCL9	Amplification of the IFN‐γ signaling	⊥	^254^
P‐10/CXCL10 & MMP‐1	Inflammation followed by chemotaxis and angiogenesis	⊥	^255^
MCP‐1/CCL2, CCL3/MIP1‐α, CCL4/MIP1‐β, CCL8/MCP2, CCL20/MDC & CCL22/MIP3‐α	Migration and infiltration of monocytes/macrophages	⊥	^256^
RANTES/CCL5	T‐ cell development and DC migration	↑	^257^
PGE2	Mediates inflammation and activates IFN‐γ	⊥	^258^
GM‐CSF	Development & maturation of Myeloid cells and differentiation of Dendritic cell	↓	^253^
IL‐1β	Cytokinemia, Hypercoagulation, disseminated intravascular coagulation, and prolonged lung infection	↓	^253^
TNF‐α	Cytokine upregulation as well as acting as an amplifier of inflammation	⊥	^253^
IL‐12	Drives NK cell and T‐cell production and also IFN‐γ production	↑	^259^
IL‐6	Induce inflammation and cytotoxic CD8^+^ T cells	⊥	^253^
IL‐2	Decrease of CD8^+^ T cells, IFN‐γ production	↑	^260^
IL‐4	Differentiation of M2 macrophages, activation of different growth factors	↓	^261^
IFN‐γ	Promoting macrophage activation and mediating host defense against pathogen infection	↑↓	^260^
IL‐10	Rapid accumulation of proinflammatory cytokines	↑	^262^
IL‐13	Induction and maintenance of IgE production and IgE‐mediated allergic responses	↓	^263^
IL‐18	Macrophage activation	⊥	^264^
TGF‐β	Recruits more neutrophils at infection sites	↑	^265^
**Curcumin**			
IL‐10	CD4+T cell differentiation	↑	^266^
IL‐1β	Recruitment of proinflammatory cytokines	⊥	^267^
IL‐18	Promoting inflammatory cytokines	↑	^268^
TNF‐α	Amplifier of inflammation	⊥	^269^
IL‐8/CXCL8	Activator of neutrophils and γδ T‐cells	⊥	^270^
MMP‐1	Chemotaxis and angiogenesis	⊥	^271^
MCP‐1/CCL2, CCL3/MIP1‐α, CCL4/MIP1‐β, CCL8/MCP2, CCL20/MDC, & CCL22/MIP3‐α	Monocyte migration and infiltration	⊥	^272^
RANTES/CCL5	T‐cell development	⊥	^272^
TGF‐β	Recruitment of more neutrophils at infection site	⊥	^273^
IFN‐γ	Promote inflammation	⊥	^274^
IL‐17	Promote inflammation	⊥	^274^
GM‐CSF	DC development	⊥	^275^
**Quercetin**			
IFN‐γ	Macrophage activation	↑	^276^
IL‐4	Macrophage activation/differentiation	↑	^276^
TGF‐β	Neutrophil recruitment	⊥	^277^
TNF‐α	Inflammatory mediator	⊥	^278^
GM‐CSF	DC maturation and development	⊥	^279^
MMP‐1	Angiogenesis and chemotaxis	⊥	^280^
IL‐1β	Promotes inflammation	⊥	^281^
IL‐18	Macrophage activation	⊥	^281^
PGE2	Promotes inflammation and activates IFN‐γ	⊥	^282^
**Vitamin‐C**			
IFN‐γ	Promoting macrophage activation	↑	^283^
IL‐4	Macrophage activation	↑	^283^
IL‐1β	Induce inflammation	⊥	^284^
TNF‐α	Mediates inflammation	⊥	^284^
IL‐10	T cell differentiation	⊥	^285^
IL‐6	Induce cytotoxic CD8^+^ T cells	⊥	^285^
GM‐CSF	Myeloid cell development	↓	^286^
MMP‐9	Chemokine activation	⊥	^285^
PGE2	Promotes inflammation	↑↓	^287^
IL‐12	Drives NK cell activation	↑	^288^

*Note*: ↑, Increase; ↓, Decrease; ⊥, Inhibition and ↑↓, mean context dependent up/down regulations.

**FIGURE 6:**
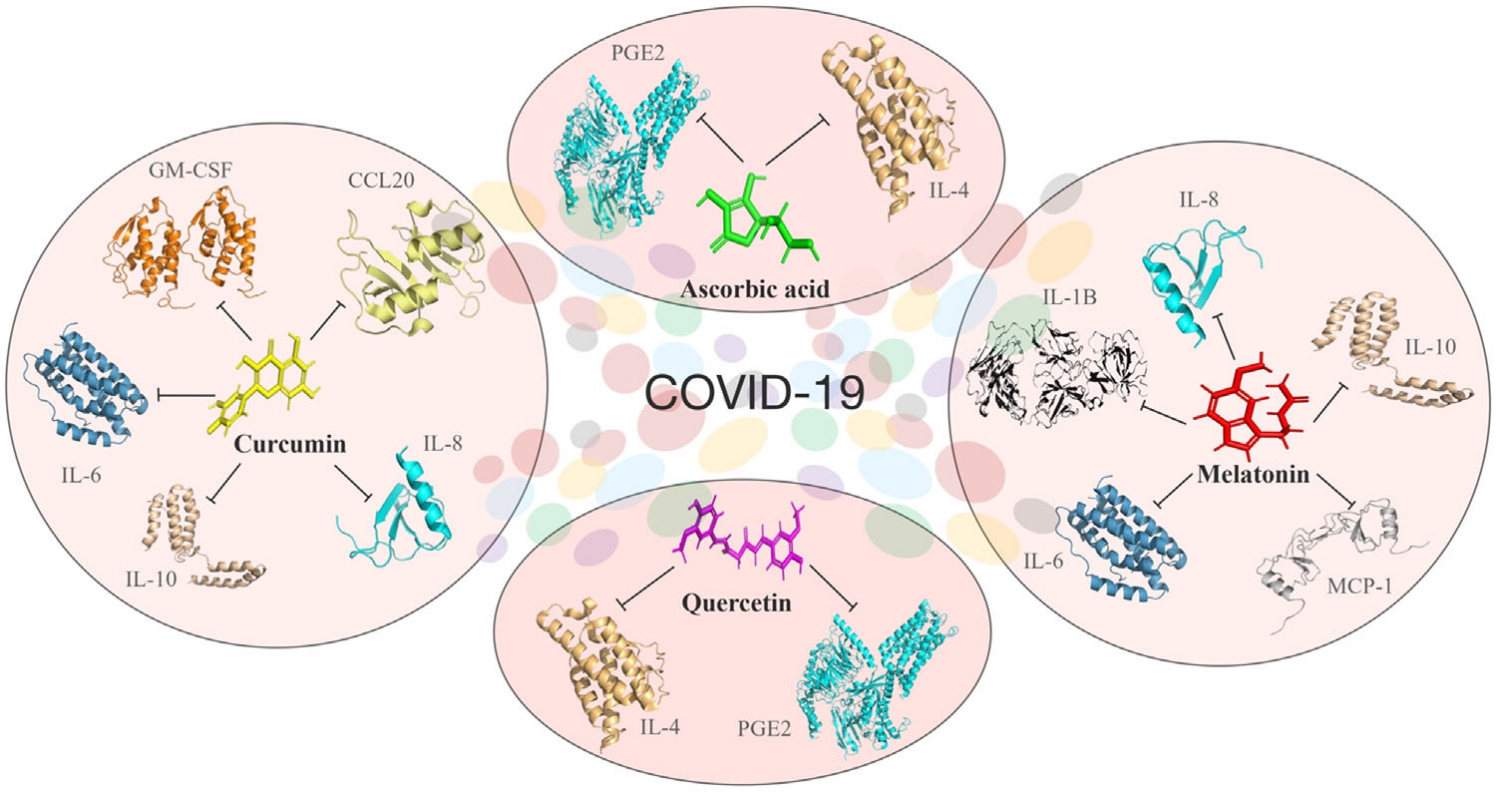
Preventive measures for coronavirus disease 2019 (COVID‐19) by antioxidants. Structure‐based mode‐of‐action of reactive oxygen species (ROS) scavengers’ melatonin, curcumin, quercetin, and ascorbic acid on pro‐/anti‐inflammatory cytokines in COVID‐19.

Many antioxidants are available to use in combination therapy for drug repurposing or repositioning strategy and started immediately after the outbreak and used to treat SARS‐CoV‐2 infected patients which represent valid and alternative procedures for providing suitable medications among the potential and previously used clinically tested compounds against viral diseases. Further repurposing of drugs is also suggested for SARS‐CoV‐2 infection as a parallel strategy.^194^ Most of the repurposed or repositioned drugs for COVID‐19 are not yet approved and ready to apply. Thus, antioxidative molecules are mostly used to protect COVID‐19 patients from neurological disorders and severe organ‐level damage by controlling various pro‐/anti‐inflammatory molecules responsible for the disease and their available structural information (Figure 6).

### 8.1 Melatonin

Melatonin (N‐acetyl‐5‐methoxy tryptamine), a bioactive molecule shows health‐nurturing properties that include treatment of sleeping disorder, delirium, atherosclerosis, respiratory diseases, and viral infections.^195^ Although melatonin is not a viricidal agent but can suppress viral infection. Recent reports indicate that it is effective against oxidative stress induced by viruses, bacteria, and radiation. It shows potent antioxidant, anti‐inflammatory and immune‐modulatory properties.^196^, ^197^ So, testing the efficiency of melatonin on the respiratory and other systems linked to SARS‐CoV‐2 is incorrigible. Binding sites and endogenous synthetic machinery of melatonin are distributed in the entire mammalian system. From several clinical reports, it has been observed that coronavirus infection is not localized in the lungs and upper respiratory tract though it primarily affects the respiratory tract later it spreads to other organs, for example, gastrointestinal systems and central nervous systems resulting in multi‐organ failure.^198^, ^199^, ^200^

Melatonin scavenges toxic molecules like peroxynitrite anion, hydroxyl and peroxyl radicals, and singlet oxygen.^201^, ^202^ It inhibits the peroxidation of membrane lipids and proteins to reduce severe inflammation. Free radical scavenging activities of this antioxidant have been observed in several studies. Its metabolites also show antioxidant properties.^203^, ^204^ As SARS–CoV‐2 infection also generates excessive ROS,^205^ we should protect COVID‐19 patients through reduction of the oxidative damages. In fact, it reduces the proinflammatory cytokines TNFα, IL‐1β, IL‐6, and IL‐8 and elevates anti‐inflammatory cytokines, for example, IL‐10. It also acts as a neuroprotective agent through its anti‐inflammatory and antioxidative properties. In brain ischemia, gastritis, and periodontitis diseases melatonin shows anti‐inflammatory actions by targeting the TLR4 receptor.^206^, ^207^ It protects neurological patients by an inhibiting cerebral inflammatory response, cerebral edema, and BBB permeability. It is naturally synthesized in the pineal gland.^208^ Synthetic melatonin is structurally similar to the endogenously produced form and is recognized as a human‐friendly agent. It shows anti‐inflammatory responses even at high doses. Lower intake of melatonin (5 mg/day for 5 days) can lower pro‐inflammatory cytokines levels in brain reperfusion and coronary artery reperfusion.^209^ Even in other human diseases it has no harmful effect even used for a longer period. The physiological level of melatonin is not enough to prevent inflammation in severe COVID‐19 patients. The dose selected for patients in the clinical trial is 8 mg/kg body weight/day and showed no harmful effects whether given in chronic or acute conditions. Thus, the safety margin of melatonin is very high and encouraged healthcare professionals to use it in patients with both cerebral ischemia and COVID‐19.

### 8.2 Curcumin

Curcumin [(1E,6E)−1,7 bis (4‐hydroxy‐3 methoxy phenyl)1,6 heptadiene‐3,5 di one] is a herbal medicine, extracted from Curcuma Longa plants, used to treat human diseases without any side effects, shows antioxidative actions even at low doses. From clinical investigations, it has been shown that curcumin is effective to reduce inflammation caused by viral infections. It has also been proven that curcumin has anti‐inflammatory, anti‐cancer, and anti‐diabetic effects in addition to its antioxidant activity. The nullifying effects on these diseases occur through modulation of immune response via inhibiting cytokine storm caused by viral infection.^210^, ^211^, ^212^ The antiviral potential of curcumin has been approved by US Food and Drug Administration due to its safety with multiple actions.^213^, ^214^ It shows protective effects against inflammatory diseases, neurological diseases, cardiovascular diseases, pulmonary diseases, metabolic diseases, liver diseases, and even cancer. Curcumin treatment can decrease the production of inflammatory mediators like cytokines, chemokines, and adhesion molecules in the brain of cerebral ischemic patients. Additionally, it attenuates glutamate neurotoxicity in the hippocampus by the suppression of ER stress‐associated Thioredoxin interacting protein (TXNIP)/NLRP3 inflammasome activation in a manner dependent on AMP‐activated Protein Kinase. It has antiviral activities against many viruses, for example, vesicular stomatitis, para influenza type 3, flock house, Herpes simplex, Zika virus, and even SARS‐CoV‐2.^215^, ^216^, ^217^ It also interacts directly with DNA polymerase, thioredoxin reductase, focal adhesion kinase, protein kinase, tubulin, and lipoxygenase. Modulation of intercellular signaling cascades by curcumin limits the viral multiplication by with interfering crucial steps in their life cycle, for example, replication and attachment.^218^ Additionally, it can block the entry of viruses by changing the conformations of surface proteins and the host cell's membrane proteins to modulate the lipid bilayer. Using molecular docking studies by Lorizate et al.,^219^ has been shown that curcumin can bind to the target receptor proteins like SARS‐CoV‐2 protease, spike glycoprotein RBD and peptidase domain of ACE2. Curcumin obstructs viral infection by preventing penetration and replication.^220^, ^221^ The positive charges of curcumin interact electrostatically with the Porcine epidemic diarrhea virus (PEDV), a member of coronaviridae, to compete for their binding with the host cell. Curcumin inhibits the ACE2 receptor for suppressing entry of SARS‐CoV‐2, shown by molecular docking studies. Additionally, during the synthesis of negative‐stranded RNA curcumin inhibit PEDV at the replication step resulting in reduced plaque numbers supporting the potential role of curcumin as a strong antiviral agent.^222^

The expression, production, and activity of IL‐10 were potentially increased by curcumin and its derivatives. In the ALI mouse model, curcumin inhibits lung injury and induces the differentiation of regulatory T cells through the upregulation of IL‐10 production.^223^ Same effect has also been observed in neuropathic models and other inflammatory diseases. Thus, curcumin can play a ‘double‐edged sword’ by downregulating inflammatory cytokines and upregulating anti‐inflammatory cytokine IL‐10. As a polyphenolic antioxidant compound, curcumin can directly scavenge ROS (superoxide radical and hydroxyl anion) by its hydroxyl group on the benzene ring. It reduces the lecithin peroxidation and oxidative damage of DNA.^224^ Furthermore, its ROS scavenging ability is indirectly playing through the upregulation of SOD‐2. SOD‐2 regulates the conversion of O^−^_2_ to H_2_O_2_ which is ultimately reduced to H_2_O by glutathione (GSH).^225^, ^226^ Curcumin also opposes the effect of ROS and expression of proinflammatory cytokines (IL‐1β and IL‐18) by downregulating thioredoxin interacting protein TXNIP/NLRP3 (NLR pyrine domain containing 3).^227^

Curcumin inhibits inflammation by regulating multiple signaling pathways engaged in peroxisome proliferator‐activated receptor γ, Jun N terminal Kinase (JNK), NF‐κβ, and Nrf2.^228^, ^229^, ^230^, ^231^ Curcumin lowers the severity of pneumonia caused by viral infection through inhibition of NF‐κβ signaling and IL‐10 production. It reduces oxidative stress and IAV viral pneumonia through the activation of Nrf2 and inhibition of TLR2/4, P38/JNK, MAPK, and NF‐κβ. Thus, curcumin has many beneficial roles to prevent diseases caused by coronaviruses.^232^ Curcumin nano micelle supplement inhibits oxidative stress‐reducing inflammatory biomarker, TNF‐α.^233^, ^234^ From the phase II randomized control study, it has been shown that the topical application of curcumin and its polyherbal cream has a higher Human Papilloma Virus clearance rate than placebo. Altogether, it has been concluded that curcumin is an attractive molecule to treat COVID‐19 patients.

### 8.3 Quercetin

Quercetin (3,3′,4′5,7‐pentahydroxyflavone) is a known plant flavonoid found in leaves, seeds, and grains of vegetables. Generally, it is linked with residual sugars to form plant glycosides.^235^, ^236^ It has been observed by different studies that quercetin has antioxidant, anti‐inflammatory, antiviral, and immune‐protective effects. It shows antiviral effects by inhibiting polymerases, proteases, and reverse transcriptase.^237^, ^238^ It also suppresses DNA gyrases and binds viral capsid proteins. It is a free radical scavenger and shows antioxidant properties in vitro and in vivo.^239^, ^240^ The anti‐inflammatory activity of quercetin is by flavonoid activity on arachidonic acid through the leukotriene‐prostaglandin pathway. The therapeutic effect of quercetin was also highlighted in ischemic brain injury as it plays a role in limiting the secretion of immunological factors by various immune cells that can inhibit thrombosis, oxidative stress, apoptosis, and autophagy. Quercetin attenuates ischemia‐reperfusion injury by protecting the BBB through Sirt1. In addition, the diversified biological activity of quercetin is proved by several studies that include inhibition of platelet aggregation, lipid peroxidation, and proinflammatory mediators like lipoxygenase and phospholipase A2.^241^, ^242^ It also affects the functions of several lipids, serine/threonine kinases and inducible NOS2 (iNOS‐2). In SARS‐CoV‐2 infection, quercetin‐3β galactoside binds to the 3CL‐pro protease domain at Gln 189. The 3CL‐pro site of SARS‐CoV‐2 is the ideal binding site for quercetin and its derivatives through their hydroxyl groups.^243^ Link Yi and colleagues said, “quercetin offers great promise as a potential drug in the clinical treatment of SARS”.^244^

### 8.4 Vitamin C

Globally approved good quality vaccines are not yet enough. Hence, throughout the globe, many therapeutic approaches have emerged. Among these vitamin C administration in critically ill COVID‐19 patients is remarkable. It is an incredibly good antioxidant, although does not show a direct lethal effect on viruses. It is one of the major nonenzymatic antioxidants present in the plasma that attenuates oxidative stress, inflammation, maintaining vasopressure, and the immune system.^245^, ^246^, ^247^ In the human body, it functions as a weak antihistamine and provides relief from flu‐like symptoms such as sneezing, a running or stuffy nose, and swollen sinuses. It can significantly reduce the incidence of pneumonia and reduce the susceptibility of lower tract viral infections, particularly at a low level in sepsis. SARS‐CoV‐2 infection was associated with higher levels of inflammation and poorer outcomes with multiple organ failure and mortality.^200^ The cytokine surge is activated after viral infections that lead to the accumulation of neutrophils in the lungs and destroy the alveolar capillaries. From early clinical studies, it has been shown that vitamin C inhibits these processes, hence definitely useful for the treatment of COVID‐19 and ischemic patients.^248^ It acts as a free radical scavenger, prevents free radical‐induced cell damage,^249^, ^250^, ^251^ and provides protection against various diseases like arthritis, atherosclerosis, cancer, diabetes, and ischemia. It decreases viral infection and pneumonia and protects people from avian coronavirus infection. Vitamin C treatment in critically ill and hospitalized patients shows mixed results on mortality.^248^, ^252^ Its application on COVID‐19 patients with ischemia provides positive results and high intravenous doses were safe.

Inflammatory mediators which are discussed in the present review as protective agents against COVID‐19‐associated neuronal damage are produced by different infiltrating immune cells such as neutrophils, lymphocytes, and monocytes. These inflammatory cells use cytokines and chemokines to coordinate all stages of inflammatory immune responses through the upregulation and downregulation of specific chemokines and cytokines (Table 1).

## 9 CONCLUSIONS AND OUTLOOK

SARS‐CoV‐2 infection generates proinflammatory processes at its central level, therefore the use of different anti‐inflammatory compounds will be required to alter the possible consequences of COVID‐19 with neurological diseases and avoid their adverse effects on multiple organs. This review will lead to a search for preventive and therapeutic strategies for treating acute conditions of the disease. Antioxidant‐based preventive therapy has not been rigorously tested yet on COVID‐19 patients. However, some previous research proved that antioxidants showed a significant increase in prophylaxis support against many viral diseases including SARS. Blocking the release of proinflammatory cytokines can be possible through the application of natural compounds as prodrugs having anti‐oxidative and anti‐inflammatory functions. Based on the pharmacokinetics and pharmacodynamics study, the natural compounds in the form of prodrugs and nanoparticulated formulations could be tested in clinical trials before implementing preventive therapies against COVID‐19, although there is a possibility of complications in the disease pathology.

Preclinical studies on patients with lung complicacy during the SARS‐CoV‐2 infection indicate that administration of N‐acetylcysteine (NAC), a precursor of GSH can be used to limit oxidative stress in lung injury as it increases the intracellular GSH content by elevating glutathione‐S‐transferase activity. NAC also decreases the levels of TNF‐α, IL‐8, IL‐6, and IL‐12 induced by the inflammatory immune response. eNOS and iNOS can also be triggered by vitamin C which results in an increase in pro‐inflammatory mediators like TNF‐α and IL‐6. So, macrophages are activated and the gathering of neutrophils takes place to counter the pathogenic situation. Quercetin basically inhibits the H^+^‐ATPase of the lysosomal membrane, therefore inhibiting the viral load, at the same time it reduces drug resistance.

Neurological damages because of COVID‐19 led us to know the clinical implications of different antioxidants. As the disease quickly spread worldwide, close clinical monitoring is essentially needed to understand the effect on the severity of symptoms and their consequences. Comorbidity conditions can also be overcome by adjuvant therapy in SARS‐CoV‐2 infection as it enhances the level of endogenous antioxidants during the treatment of COVID‐19. Nevertheless, more studies are still needed in clinical trials based on the conditions of patients under treatment to emphasize the benefits of antioxidant supplementation along with steroid‐based therapy for the treatment of COVID‐19.

## AUTHOR CONTRIBUTIONS

Conceived the idea and review structure: SS, SK, MB, and MKG; Wrote the manuscript: SS, SK, and MKG; Material collection and editing: SS, SK, MB, PG, and MKG. All authors have read, agreed to the final draft, and approved the manuscript.

## CONFLICT OF INTEREST STATEMENT

The authors declare no conflict of interest.

## ETHICS STATEMENT

This article does not contain any studies with human participants or animals performed by any of the authors.

## Data Availability

Not applicable.
